# The application of bronchoscopy in the assessment of immune checkpoint inhibitor-related pneumonitis severity and recurrence

**DOI:** 10.1038/s41598-024-66768-6

**Published:** 2024-07-25

**Authors:** Cuiyan Guo, Qi Zhang, Peining Zhou, Yuan Cheng, Ligong Nie, Guangfa Wang

**Affiliations:** Department of Respiratory and Critical Care Medicine, Peking University First Hospital, NO.8 Xishiku Street, Xicheng District, Beijing, 100034 China

**Keywords:** Bronchoscopy, Checkpoint inhibitors pneumonitis, Recurrence, Severity, Biopsy, Cancer therapy, Lung cancer

## Abstract

To explore the role of bronchoscopy for the assessment of checkpoint inhibitor pneumonitis (CIP), a retrospective single-center study was conducted to assess patients diagnosed with CIP at grade 2 or above and also underwent bronchoscopy between January 2020 and December 2022. Clinical data and bronchoscopic findings were recorded. The treatment data and prognosis information were collected. Twenty-one patients who underwent bronchoscopy and were diagnosed with CIP were enrolled in this study. All patients underwent bronchoalveolar lavage fluid (BALF) analysis. Of them, T lymphocyte subsets of BALF were tested in 15 cases. Transbronchial cryobiopsy (TBCB) was performed in 8 patients, and transbronchial lung biopsy was performed in 5 patients. 3 patients developed pneumothorax after TBCB and all recovered without serious compilations.14 patients experienced grade 2 CIP, while 7 patients ≥ grade 3 CIP. Symptoms were improved in 19 (90.5%) patients after standard treatment adhering to CIP guidelines. However, 5 patients relapsed during steroid tapering. Factors related to the severity and recurrence of CIP were analyzed. Patients with previous interstitial lung disease (ILD) were more likely to develop high grade CIP than those without [83.3% (5/6) versus 15.4% (3/15), P = 0.011].The odds ratio (OR) was 32.5 (95% CI 2.284–443.145, P = 0.009). Increased BALF lymphocyte percentage was associated with high grade CIP, OR 1.095 (95% CI 1.001–1.197, P = 0.047), and higher possibility of CIP relapse, OR 1.123 (95% CI, 1.005–1.225, P = 0.040). Lymphocyte subsets were tested in 15 patients. CD4/CD8 > 1 was found in 80% (4/5) of relapsed patients and 20% (2/10) of patients without relapse (P = 0.047). The OR was 16.00 (95% CI 1.093–234.24, P = 0.043). In this retrospective study, patients with previous ILD was more likely to develop high grade CIP. Higher lymphocyte percentage in BALF was associated with high grade CIP and susceptibility to relapse during treatment of CIP. A CD4/CD8 ratio greater than 1 in lymphocyte subsets of BALF was associated with higher possibility of CIP relapse. We found that TBCB is a safe procedure in CIP patients.

## Introduction

Immune checkpoint inhibitor (ICI) therapy has revolutionized the treatment of advanced malignancies, and is increasingly applied to various cancers. However, ICI related toxicities, termed immune-related adverse events (irAEs), also pose significant risks to patient’s wellbeing and greatly burden the health system. CIP is a relatively common but potentially life-threatening adverse event, and also impacts subsequent anti-tumor treatment. When adjusted for guaranteed time bias, the development of CIP worsens survival in non-small cell lung cancer (NSCLC) patients^1^.

In the current irAE guidelines^2^, bronchoscopy is recommended for CIP of grade 2 or above. It is generally accepted that the role of bronchoscopy in CIP is to exclude infections and tumor infiltration^2^.Nevertheless, the further value of bronchoscopy in the management of CIP has not been well explored. Specimens obtained by bronchoscopy, including liquid and tissue samples, can be used for further examination. Persistent BALF lymphocytosis is associated with development of chronic CIP and may be an indicator for need of long-term immunosuppression of more than 12 weeks^3^. The proportions of CD8+ T cells positive for both PD-1 and T cell immunoglobulin and mucin domain-containing protein 3 (TIM3) or T cell immunoreceptor with Ig and ITIM domain (TIGIT) in BALF were positively correlated with the grade of ICI-related ILD^4^. Wang et al.^5^ found that crosstalk between pathogenic Th17/Th1 cells and pro-inflammatory monocytes in BALF, and activation of Th17(/Th1)/IL-17A (/IFN-γ) pathways may play a key role in the pathogenesis of CIP. These results indicate that T cell subgroups found in BALF may provide insight into the development mechanisms of CIP. Therefore, the specimens, which were collected directly from the lung injury sites, could precisely reflect the immune microenvironment and its intrinsic characteristics. Further research is needed to elucidate the specific mechanisms and confirm the role of bronchoscopy in CIP management.

The purpose of this study was to assess the role of bronchoscopy in CIP patients, and the safety of biopsy, especially transbronchial cryobiopsy (TBCB) in CIP. The severity, recurrence, and overall survival were also studied.

## Methods

Patients with clinically suspected CIP were enrolled between January 2020 and December 2022. This retrospective single-center study was approved by the Ethics Committee of Peking University First Hospital (2023–139).

The diagnostic criteria of CIP was: a) previous use of ICI; b) new-onset pulmonary infiltration (e.g., ground glass opacities, patchy consolidation, thickened interlobular septa, traction bronchiectasis, and reticulonodular changes); c) pulmonary infections, tumor progression, or interstitial lung disease of other causes could be ruled out. The severity of CIP was graded as follows: G1 Asymptomatic; confined to one lobe of the lung or < 25% of lung parenchyma; G2: Symptomatic; Involves more than one lobe of the lung or 25–50% of lung parenchyma; G3: Severe symptoms; Hospitalization required: Involves all lung lobes or > 50% of lung parenchyma; limiting self-care activity of daily living; G4: Life-threatening respiratory compromise^2^.

### Patients

Inclusion criteria

- Patients underwent bronchoscopy with suspected CIP of grade 2 or above.
- Previous use of ICI.
- Lung injury unattributable to other factors.

Exclusion criteria

- Severity of CIP less than grade 2.
- Patients lost to follow-up or without sufficient follow-up data.
- Pulmonary infection could not be ruled out.

### Bronchoscopy procedures

All bronchoscopy procedures followed routine examination process. Bronchoalveolar lavage fluid (BAL), with or without transbronchial lung biopsy (TBLB), was performed under conscious sedation. TBCB was performed via rigid bronchoscopy under general anesthesia. Bedside ultrasound and/or chest X-ray were conducted post-procedure to check for pneumothorax.

Bronchoalveolar lavage was performed as follows. When the bronchoscope reached the target segment, sequential 20 ml aliquots of normal saline totaling 100–120 ml were injected and then aspirated for analysis. The samples were sent for bacterial, viral, fungal, tuberculosis-related tests, metagenomics Next Generation Sequencing (mNGS), and cell differential counts and lymphocyte subsets. Tissue specimens, usually 4–6 TBLB specimens or 2–3 TBCB specimens, were sent to pathologic diagnosis.

### Clinical information

The clinical information was retrospectively recorded, including demographic characteristics, symptoms, laboratory tests, Chest CT manifestations, patient management and prognosis.

### Statistics methods

Statistical analysis was performed using the SPSS 22.0 statistical Software package. The chi-square test or Mann–Whitney U test were used for comparison between groups, and the Fisher’s exact test was used when necessary. Univariate logistic regression analysis was used to determine the independent risk factors directly related to severity and recurrence of CIP. Kaplan–Meier curves were used for analysis of factors affecting overall survival.

### Ethical statement

The study was reviewed and approved by the Peking University First Hospital Institutional Review Board (IRB), approval number 2023–139. The need for informed consent was waived by the Peking University First Hospital IRB. Data collection was performed in accordance with relevant guidelines and regulations of the committee. The study adhered to the ethical principles outlined in the Declaration of Helsinki for experiments involving human beings.

## Results

**Figure 1:**
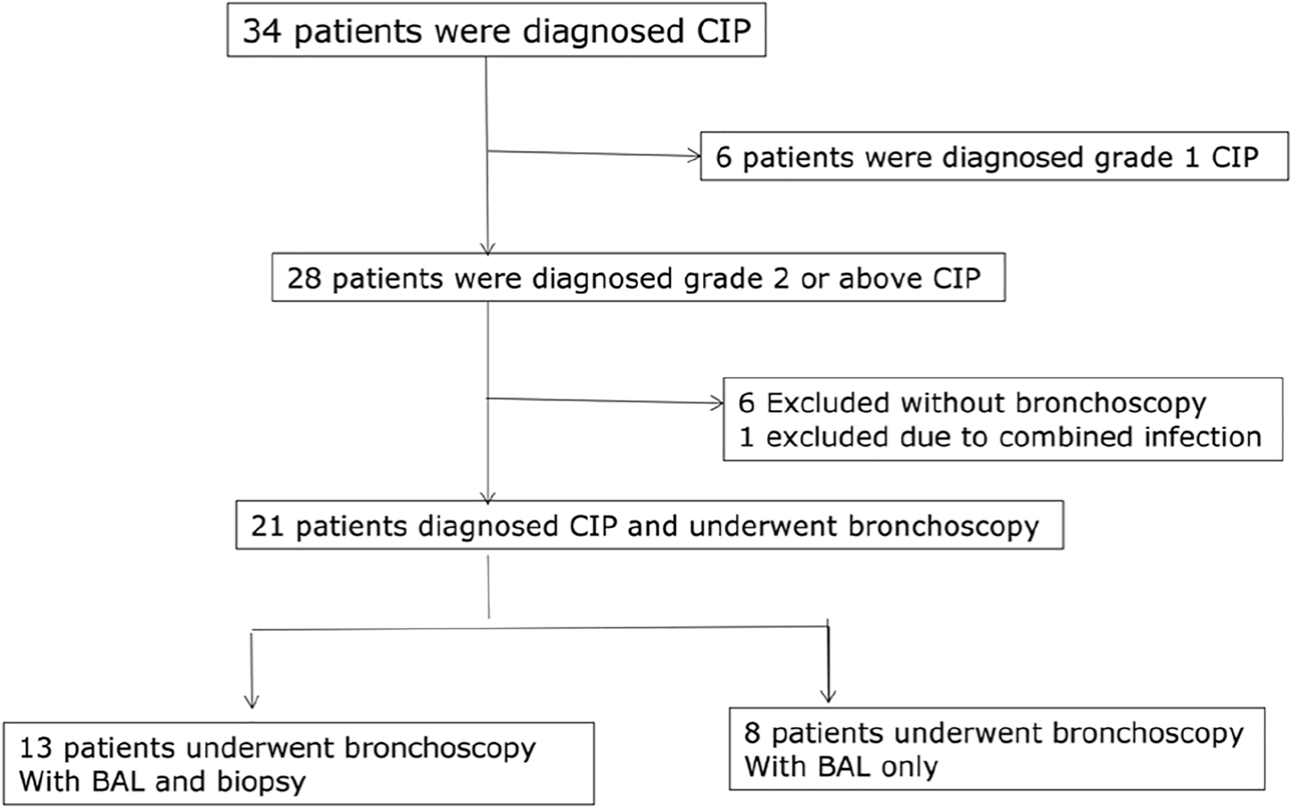
Study flowchart.

34 patients were diagnosed with CIP from July 2020 to December 2022 at Peking University First Hospital. Among them, 6 presented with grade 1 CIP and improved after discontinuation of immunotherapy. 6 patients were excluded due to the absence of bronchoscopy and 1 patient was excluded due to concurrent Covid-19 infection. Ultimately, 21 patients were included in this study, with negative mNGS and culture of BALF. Figure 1 shows the flowchart of the overall screening process.

Baseline demographic characteristics are shown in Table 1. The median age was 61 years old. The majority were male (85.7%). Among 15 (71.4%) smokers, 13were current smokers and 2 past smokers. There were 11 cases of lung cancer, 3 Renal cell carcinoma, 2 bladder cancer, 1 esophageal cancer, 1 gastric cancer, 1 endometrial cancer, 1 head and neck squamous cell carcinoma, and 1 colon cancer. Among the 21 enrolled cases, PD-1 inhibitors were used in 16 (76.2%) cases. The median cycle of immunotherapy before the onset of CIP was 4 cycles and the median time from the onset of symptoms to diagnosis was 2 weeks. First-line immunotherapy was used in 17 (81.0%) patients. 9 (42.9%) patients received thoracic radiotherapy before the onset of CIP.

**Table 1:** Baseline clinical characteristics.

N = 21	
Age (years), median (IQR)	61 (57–69.25)
Male	18 (85.7%)
Smoking status	
Never	6 (28.6%)
Yes (current/past)	15 (71.4%) (13/2)
Thoracic radiotherapy	
Yes	9 (42.9%)
No	12 (57.1%)
PD-1 inhibitor	16 (76.2%)
PD-L1 inhibitor	5 (23.8%)
Treatment line	
1	17 (81.0%)
≧2	4 (19.0%)
Bronchoscopy results	
BALF	
Lymphocyte percentage (N = 19*)	
≧20%	14 (73.7%)
< 20%	5 (26.3%)
CD4/CD8 ratio (N = 15^#^)	
≧1	6 (37.5%)
< 1	9 (62.5%)
Pathology (N = 13)	
OP	5 (38.4%)
AFOP	3 (23.1%)
NSIP	3 (23.1%)
DAD	2 (15.4%)

*Of the 21 patients who underwent bronchoscopy, BALF differential cell count was performed in 19 patients.

^#^Lymphocyte subset testing was performed in 15 patients.

Cell differential count of BALF was performed in 19 patients, and lymphocyte subsets were tested in 15 cases. 13 patients underwent lung biopsy, 8 TBCB and 5 TBLB.

### Factors associated with the severity of CIP

Of the 21 patients enrolled, there were 14 patients presenting with grade 2 CIP (defined as low-grade group), 7 presented with grade 3 or higher CIP (defined as high-grade group). Table 2 shows factors associated with the severity of CIP. The median age was 60 years old in the low-grade group and 65 years old in the high-grade group. Patients with previous ILD were more likely to develop high-grade CIP, compared with patients without previous ILD, 83.3% (5/6) versus 15.4% (2/15), respectively (P = 0.006). No correlation was found between severity of CIP and smoking status, thoracic radiotherapy history, treatment line or tumor type. The median neutrophil lymphocyte ratio (NLR) was higher in the high-grade group than in the low-grade group, 6.33 versus 3.57, respectively (P = 0.025). Cases in the high-grade group had lower lymphocytes and higher eosinophils, neutrophils, LDH and high-sensitivity CRP (hsCRP), though no statistically significant difference was reached.

**Table 2:** Factors associated with CIP severity.

	Grade 2 (N = 14)	Grade ≧3 (N = 7)	P
Age (years), median (IQR)	60 (56.75–66.25)	65(54–72)	0.369
Smoking status			
Never (N = 6)	5 (35.7%)	1(14.3%)	0.614
Thoracic radiotherapy (N = 9)	6 (42.9%)	3 (42.9%)	0.676
Pre-ILD			
Yes (N = 6)	1(7.1%)	5(71.4)%	0.006
PD1inhibitor (N = 16)	11(78.6%)	5 (71.4%)	0.557
Duration of immunotherapy			
< 3 months (N = 12)	6 (42.9%)	6 (85.7%)	0.078
LDH, median (IU/L) (IQR)	202.5 (171.25–236.35)	240.0 (162.0–364.0)	0.191
hsCRP, median (mg/L) (IQR)	23.9 (8.46–81.5)	51.0 (33.44–78.53)	0.452
Blood routine test			
Lymphocyte, median (K/mcL) (IQR)	0.98 (0.74–1.50)	0.60 (0.60–1.0))	0.167
Eosinophil, median (K/mcL) (IQR)	0.1 (0.0–1.480)	0.5 (0.1–2.3))	0.246
Neutrophil, median (K/mcL) (IQR)	3.67 (2.33–4.08)	4.7 (3.2–7.7)	0.218
NLR, median (IQR)	3.57 (2.30–5.77))	6.33 (5.35–9.63)	0.025
BALF			
Lymphocyte percentage, median (IQR)	21.50 (11.75–29.75)	56.0 (25.5–64.0))	0.032
CD4/CD8 ≧1 (%) (N = 15)	3 (30%)	3(60%)	0.329
Pathology (N = 13)			
OP (N = 5)	3	2	
AFOP (N = 3)	2	1	
NSIP (N = 3)	3	0	
DAD (N = 2)	2	0	
Survival rate (N = 15) (%)	11(78.6%)	4(57.1%)	0.299

As for the cell differential of the BALF, the median lymphocyte percentage was higher in the high-grade group than low-grade group, 56.0% versus 21.5%, respectively (P = 0.032). There was no significant correlation between BALF lymphocyte subsets and the severity of CIP. Table 3 shows binary logistic regression analysis of univariate factors related to the grade of CIP. Patients with previous ILD were more likely to develop high-grade CIP than patients without previous ILD (OR = 32.5, 95% CI 2.284–443.145, P = 0.009). Higher BALF lymphocyte percentage was associated with higher possibility of high-grade CIP (OR = 1.095, 95% CI 1.001, 1.197, P = 0.047).

**Table 3:** Logistic regression analysis of risk factors for high-grade CIP.

Factors	Univariate analysis		
	OR	95% CI	P
Age	1.059	0.932–1.202	0.380
Sex (male vs. female)	1.00	0.075–13.367	1.00
Smoking status (yes vs. never)	3.333	0.308–36.110	0.322
Thoracic radiotherapy (yes vs.no)	1.00	0.106–6.255	1.00
Pre-ILD (yes vs.no)	32.5	2.284–443.145	0.009
Duration of immunotherapy (< 3 months vs.≧ 3 months)	0.125	0.012–1.333	0.085
PD-1 vs. PD-L1 inhibitors	1.467	0.184–11.718	0.718
LDH	1.011	0.997–1.0265	0.135
hsCRP	1.004	0.984–1.025	0.689
Blood routine test			
Lymphocyte	0.132	0.009–1.995	0.144
Eosinophil	1.439	0.679–3.050	0.342
Neutrophil	1.537	0.903–2.614	0.113
NLR	1.248	0.950–1.641	0.112
BALF			
Lymphocyte percentage (N = 19)	1.095	1.001–1.197	0.047
CD4/CD8 ≧1 vs. < 1 (N = 15)	3.50	0.372–32.971	0.274

No relationship was found between pathological type and severity of CIP. In the high-grade group, only 3 cases underwent biopsy, with 2 cases of organizing pneumonia (OP) and 1 acute fibrinous and organizing pneumonia (AFOP). In the low-grade group, 10 cases underwent biopsy, with 3 cases of OP, 2 AFOP, 3 nonspecific interstitial pneumonia (NSIP), and 2 diffuse alveolar damage (DAD).

### Risk factors for recurrence of CIP

After diagnosis, glucocorticoids were administered in 20 cases while 1 patient improved merely by ICI cessation. The follow-up chest CT was performed 1 month after steroid initiation or CIP diagnosis and then every 2–3 months. Among the 20 cases receiving steroid treatment, CIP showed rapid improvement in 18 patients, while 2 deteriorated despite timely and adequate treatment with steroids. Of the 2 steroid-resistant patients, one received infliximab but still progressed and eventually died, while the other received tocilizumab and clinically improved.

During steroid tapering, 5 (23.8%) patients experienced CIP recurrence, while 16 (76.2%) patients reported successful tapering without CIP recurrence. The median time to recurrence was 2 months after the administration of steroids. Median steroid dose at relapse was 10 mg. CIP recurrence was treated by increasing steroid dosage in combination with azathioprine in 2 patients, and increasing steroid dosage was given to 4 patients without adding any immunosuppressive agents.

Factors associated with CIP recurrence are shown in Table 4. The median age in the CIP recurrence (R-CIP) and non-recurrence groups was 54 and 61 years old, respectively (P = 0.431). The LDH, hsCRP, lymphocyte, neutrophil, eosinophil count, and NLR had no significant difference between the two groups. The median BALF lymphocyte percentage was higher in the recurrence group than the non-recurrence group, 56.0% versus 20.0%, respectively (P = 0.012). CD4/CD8 ratio ≧1 was found in 80% (4/5) of patients in the recurrence group but only 20% (2/10) in the non-recurrence group (P = 0.047). There was no significant difference in recurrence rate or CD4/CD8 ratio between different steroid regimen groups.

**Table 4:** Factors associated with the recurrence of the CIP.

	Recurrence N = 5	Without recurrence N = 16	P
Age (years), median IQR	54.0 (50.0–71.0)	61.0 (58.25–68.75)	0.431
Smoking status			
Yes (%)	4 (80%)	11(68.75%)	0.550
Thoracic radiotherapy			
Yes (%)	2 (40%)	7 (43.8%)	0.647
Pre-ILD			
Yes (%)	2 (40%)	3 (25%)	0.450
PD1 inhibitor (N = 16)	4 (80%)	12 (75%)	0.662
Duration of immunotherapy			
< 3 months (N = 12)	4 (80%)	8 (50%)	0.258
LDH (IU/L), median (IQR)	240.0 (169.0–298.0)	211.5(160.75–236.50)	0.509
hsCRP (mg/L)	50.67 (12.15–75.0)	43.6 (10.05–80.55)	0.694
Blood routine test			
Lymphocyte, median (K/mcL) (IQR)	0.60 (0.38–0.93)	0.98(0.68–1.30)	0.057
Eosinophil, median (K/mcL) (IQR)	0.13(0.055–2.90)	0.15(0.00–1.78)	0.380
Neutrophil, median (K/mcL) (IQR)	3.53(2.16–6.20)	3.83(3.08–5.65)	0.563
NLR, median (IQR)	6.0 (3.47–11.46)	3.91 (2.42–6.17)	0.409
BALF			
Lymphocyte percentage, median (%) (IQR)	56.0 (30.0–64.0)	20.0(11.75–28.0)	0.012
CD4/CD8 ≧1 (%) (N = 15)	4(80%)	2(20%)	0.047
Pathology (N = 13)			
OP (N = 5)	2	3	
AFOP (N = 3)	1	2	
NSIP (N = 3)	0	3	
DAD (N = 2)	0	2	
Survival rate	60% (3)	75% (12)	0.450

Table 5 shows binary logistic regression analysis of univariate factors related to recurrence of CIP. Higher BALF lymphocyte percentage was associated with higher possibility of CIP recurrence (OR = 1.123, 95% CI 1.005, 1.255, P = 0.040). Patients with BALF lymphocyte CD4/CD8 ratio ≧1 were more likely to develop recurrence than those with CD4/CD8 ratio < 1 (OR = 16.00, 95% CI 1.093,234.248, P = 0.043). Due to limited sample size, multivariate factor regression analysis was not conducted.

**Table 5:** Logistic regression analysis of risk factors for recurrence of CIP.

Factors	Univariate analysis		
	OR	95% CI	P
Age	0.942	0.817–1.085	0.404
Sex (male vs. female)	0.571	0.041–8.049	0.678
Smoking status (yes vs. never)	1.818	0.160–20.714	0.630
Thoracic radiotherapy (yes vs.no)	1.167	0.151–9.006	0.882
Pre-ILD (yes vs.no)	2.00	0.241–16.612	0.521
Duration of immunotherapy (< 3 months vs.≧ 3 months)	4.00	0.363–44.113	0.258
PD1 vs. PDL1 inhibitors	1.333	0.113–15.704	0.819
LDH	1.001	0.990–1.012	0.842
hsCRP	0.994	0.970–1.018	0.602
Blood routine test			
Lymphocyte	0.036	0.001–2.056	0.107
Eosinophil	1.328	0.609–2.896	0.475
Neutrophil	0.908	0.545–1.514	0.711
NLR	1.149	0.882–1.496	0.303
BALF			
Lymphocyte percentage	1.123	1.005–1.255	0.040
CD4/CD8 ≧1 vs. < 1	16.00	1.093–234.248	0.043

As for CIP histologic features (Table 4), in the recurrence group, lung biopsy was performed in 3 patients, with 2 OP and 1 AFOP. Whereas in the non-recurrent group, of the 10 patients who underwent biopsy, 3 presented as OP, 2 AFOP, 3 NSIP and 2 DAD. Due to the limited sample size, no analysis of variance was performed.

### Prognosis

The average duration of steroid therapy was 10.7 weeks and average duration of follow up was 16 months. Only one patient had persistent ILD but did not require oxygen. One patient still required oxygen when discharge, and died 1 month after discharge. No patient needed long-term oxygen.

**Figure 2:**
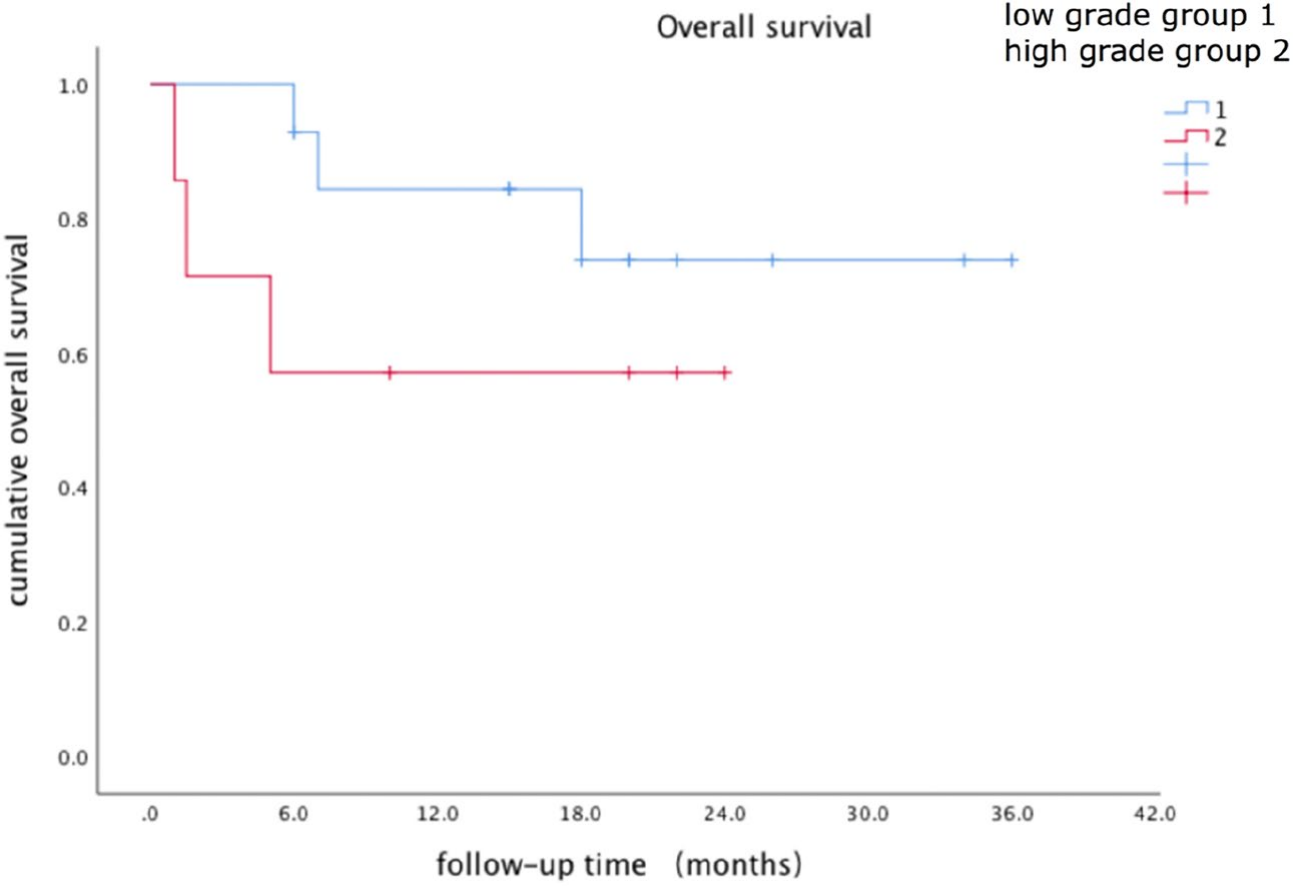
Cumulative survival rate of the low-grade and high-grade group since the onset of CIP.

**Figure 3:**
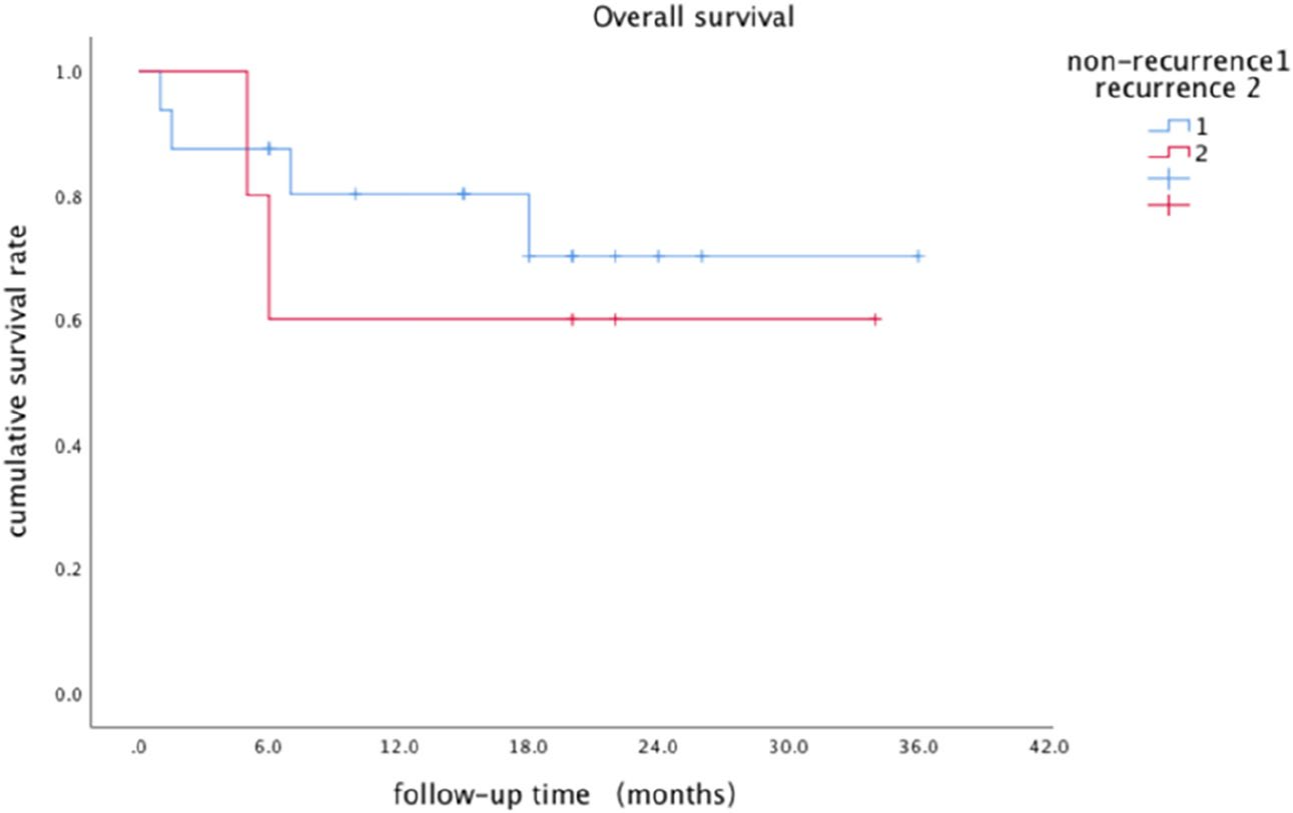
Cumulative survival rate of the recurrence group and non-recurrence group since the onset of CIP.

The Kaplan–Meier curves of the low-grade CIP group and high-grade CIP group are shown in Fig. 2. At the time of data collection, 6 patients had died, 1 of Grade 4 CIP, 1 of infection, and 4 of disease progression. By the end of the follow-up period, a trend toward a survival benefit in the low-grade group could be seen in the survival curves, however, the difference did not reach statistical significance (P = 0.219). The median survival had not yet been reached. The Kaplan–Meier curves of the recurrence group and non-recurrence group are shown in Fig. 3. The survival time had no significant difference between the recurrent group and non-recurrent group (P = 0.596). And the median survival had not been achieved by the end of follow-up.

### Safety of lung biopsy

There were 8 patients in this study who underwent TBCB. 3 developed pneumothorax after TBCB, and 2 required closed thoracic drainage. All the 3 patients recovered well without sequela. There were no procedure-related mortalities, prolonged air leak, severe bleeding, acute exacerbation, respiratory failure, or respiratory infection. Five patients in this study underwent TBLB, none had pneumothorax or hemorrhage that required intervention. Among the 13 patients who underwent lung biopsy, 2 patients died due to disease progression. The remaining 11 patients are still alive and in follow-up.

## Discussion

With the widespread use of immune checkpoint inhibitors arises the growing occurrence of immune-related adverse events. Among them, CIP is one of the most common and serious adverse events, which significantly affects patient quality of life and survival. Research on the management of CIP is still lacking. Currently, the role of bronchoscopy in CIP management is mainly for differential diagnosis, such as excluding infection and tumor progression. Osamu Nishiyama et al.^6^ systemically detailed BAL and histological findings, and correlated these findings with radiographic findings in NSCLC patients with CIP. However, there were few studies on the relationship between bronchoscopy testing and disease severity, recurrence, and prognosis^5,6^, and so far we have not found any reports on the application of TBCB in the management of CIP.

In this retrospective study, we found that in patients with previous ILD and higher BALF lymphocyte percentage were more likely to have high-grade CIP. Higher BALF lymphocyte percentage and a CD4/CD8 ratio greater than 1 in BALF lymphocyte subsets were associated with a susceptibility to relapse during treatment of CIP. TBCB in CIP is safe and feasible in general. Pneumothorax is a common complication of TBCB, but can be relieved after proper management. Necessity of TBCB should be decided on a case-by-case basis.

The overall incidence of grade 3 or higher CIP is estimated to be 1.1 to 3.2%^7,8^. Patients with CIP grade 3 or higher have a high mortality rate and poor prognosis^9,10^. However, the risk factors for severe CIP are not well recognized. Previous ILD has been reported as a risk factor for severe CIP in many retrospective literatures^9,11,12^, which is accordance with our study. In particular, there were 3 cases presenting as grade 4 CIP, all with previous ILD. 2 of the 3 patients died shortly after the onset of CIP, from CIP and secondary infection during CIP treatment, respectively. Other factors included poor performance status (PS) status^9^, emphysema^13^, previous thoracic radiotherapy^13,14^ especially those who have had obstructive pneumonia or high levels of sialylated carbohydrate antigen KL-6 levels. However, in our study, no relationship was found between thoracic radiotherapy and CIP grade. One possible reason was that ICI use concurrent with radiotherapy had a higher likelihood of high-grade CIP than sequential use^15^. Higher lung V20 (volume of normal lung receiving > 20 Gy radiation) was also associated with occurrence of high grade of CIP^16^. However, in this study, all 9 patients had ICI use after thoracic radiotherapy, 3 of which had no documented lung V20. Another reason may be that some of the cases did not have lung cancer, but rather cancer from other organs metastasized to the lung, and radiotherapy was applied to treat the lung metastases. The risk of CIP during ICI treatment may vary among tumors of different origins. For instance, the incidence of CIP was much higher in lung cancer patients than in melanoma patients^17^. The underlying mechanism of this phenomenon is still unknown.

Aiben Huang et al.^18^ found that early-onset CIP cases (onset within 6 weeks of ICI use) presented with higher-grade CIP than late-onset CIP cases. In our study, 85.7% (6/7) of high-grade CIP occurred within 3 months after ICI delivery, higher than the 42.9% (6/14) of grade 2 CIP that occurred within 3 months, though the difference did not reach statistical significance. Nevertheless, closer monitoring is still necessary to be vigilant for the occurrence of severe CIP during the initial period of ICI treatment, especially within 3 months. After all, in some severe patients, it is difficult to curb the deterioration of the disease even with high-dose steroids and immunosuppressive agents.

Research on risk factors for CIP recurrence is also scarce. The reported CIP recurrence rate ranged from 14 to 32.1%^3,19,20^. In this study, the recurrence rate was 23.8% (5/21), consistent with prior reports. Recurrence of CIP could lead to delay of anti-tumor treatment, and furthermore, prolonged use of immunosuppressive treatment increases risk of infection, seriously affecting the quality of the life. Therefore, identifying the risk factors for recurrence may be helpful for managing CIP. The reported risk factors for CIP recurrence included male sex, squamous histology, concurrent chest radiotherapy, and a shorter duration of prednisolone equivalent dose ≥ 15 mg/day^19^. None of the above clinical factors were found to be associated with CIP recurrence in this study.

As for the role of BALF testing in CIP patients in this study, higher lymphocyte percentage in BALF before treatment may be associated with high-grade CIP and may indicate a greater likelihood of recurrence. Significant lymphocytosis has been found in the pulmonary tissues and BALF from patients with CIP^21^. Enhanced and/or targeted T-cell activity against cross-antigens shared between tumor and normal tissues may result in irAEs^22^. ICI-pneumonitis may be induced by ICI-activated T cells recognizing self-peptides or epitopes shared between the tumor and self-tissue^23^. Naidoo et al.^3^ reported persistent BALF lymphocytosis in CIP despite steroid therapy may be associated with chronic ICI pneumonitis. It seemed that BALF lymphocytosis in the BALF may be involved in the development of CIP. In this study, BALF lymphocytosis was associated with higher risk of relapse, which may indicate that lymphocytosis could promote the development of CIP. Some reports have suggested that recurrent CIP may represent a distinct population different from patients with a single episode of acute ICI pneumonitis. Recurrent CIP was reported to be similar to the natural history of refractory cryptogenic organizing pneumonia (COP)^19^. The association between BALF lymphocytosis and high-grade CIP and CIP recurrence raises an interesting question worth further investigation on whether longer steroid regimens should be recommended for CIP with BALF lymphocytosis. Based on this study, longer duration of steroid therapy in patients with higher lymphocyte count may be necessary.

Although the number of cases was limited, it could be speculated that BALF lymphocyte subsets with CD4/CD8 ratio more than 1 in CIP patients may indicate a susceptibility to recurrence. It is generally acknowledged that lymphocyte subsets play different roles in the body's immune system. Higher values of CD4+ T-lymphocytes, an indicator of the human immunity, suggest better immunity. On the other hand, persistently high levels of CD8+ T-cells indicate abnormal immune activation, which means that CD4 T-cell levels may decline further and immune function reconstruction may be poor^24^. In NSCLC, Suresh et al.^25^ noted that CD4+ T cells predominated in the BAL of patients with CIP, especially CD4+ central memory subsets (Tcms, CD4+ CD45RA–CD62L+). Tcms have been shown to be more resistant to steroid-induced apoptosis than other conventional T cells. These results may be consistent with our study in that more CD4 T cells may be associated with a higher possibility of relapse. Also, circulating and lung Th17 cells contribute to pulmonary fibrosis and inflammation. An imbalance of circulating Th17 cells and regulatory T (Treg) cells was associated with the deterioration of pulmonary injury^26^. It was also reported that the conditions of sarcoidosis patients deteriorated with elevated CD4/CD8 ratios during the follow-up period, along with activated T cells present^27^. Whether the role of lymphocyte subsets in CIP is similar to that in sarcoidosis requires further study. Larger sample size studies are needed to confirm this result, and to find an applicable cutoff value for predicting high-grade CIP and recurrence. In the future, the application of flow cytometry in BALF lymphocyte subsets or histology specimens is promising for the recognition and management of CIP.

It is increasingly accepted that TBCB plays an important role in the diagnosis and management of diffuse lung diseases (ILDs)^28^. TBCB can even substitute for surgical lung biopsy in certain cases. However, the role of TBCB in CIP has not been recognized. The main reported complications of TBCB are hemorrhage and pneumothorax^29,30^. In this study, TBCB did not increase the risk of hemorrhage. Three patients (37.5%) developed pneumothorax after TBCB, which was higher than the reported pneumothoraxes of TBCB in ILDs (13.5–20.2%)^31,32^, but only two required closed thoracic drainage and were not life-threatening. Whether TBCB has a higher risk of pneumothorax in CIP than other ILDs needs to be verified future. Viktor H Koelzer et al.^33^ pointed that histopathological specimens were an important measure of quality control and may identify clinically unapparent irAEs in patients treated with immunotherapy. The application of histopathological findings in the recognition and management of ICI related adverse effects has been reported in other organs^34,35^. However, due to the limited number, the grade of inflammatory infiltration and internal features of the histological type have not been analyzed, and it is difficult to draw a relationship between the type of pathology and treatment options or prognosis. According to this study, it could be inferred that the application of TBCB in CIP is safe and tolerable, though the value of biopsy in the management of CIP remains unclear. Further implementation of lung biopsy in the management and understanding of CIP needs to be explored and verified.

### Limitations

This was a single-center retrospective study with a small number of cases and hence this can lead to sample bias. The different types of primary malignant tumors may also affect the results, due to different incidence of CIP. Also, BALF differential cell counts were tested in 19/21 cases and lymphocyte subsets in only 15/21 cases, which may affect the quality of the evidence. Multivariate factor regression analysis not conducted due to the limited number of patients in this study. Nevertheless, there have been few reports on the relationship between BALF lymphocyte percentage, lymphocyte subsets and CIP severity or recurrence^3^, and there is a lack of reports on the application of TBCB in CIP. This study lays the foundation for further research.

## Conclusion

In this retrospective study, we found that previous ILD was more likely to develop high-grade CIP. Higher BALF lymphocyte percentage was also associated with high-grade CIP and susceptibility to relapse during treatment of CIP. A CD4/CD8 ratio greater than 1 in BALF lymphocyte subsets was associated with higher possibility of relapse of CIP. We also found that TBCB is a safe procedure in CIP patients.

## Data Availability

The data of the current study are available from the corresponding author on reasonable request.
